# Military Preparedness

**Published:** 1915-10

**Authors:** 


### Military Preparedness.

While we feel that every periodical published in the U. S. should observe both the letter and the spirit of neutrality, we also feel that certain facts should be squarely faced. No sane person can imagine that in the event of war with any first-class nation, we are prepared for adequate defense. The events of the last year or so have abundantly proved that the prophecy of universal peace is unreliable. The developments and applications of science have rendered it impossible for any nation in the world to consider itself protected by isolation. The fact that we are not commercially isolated has already produced diplomatic friction of a degree that has, in the past, actually led to war. It has been predicted that we must soon or ultimately fight whichever group of nations wins the present war. Without accepting this opinion, it is possible that diplomatic friction, past and future, complicated with the antagonistic claims for loyalty between mother or ancestral country and that of residence, and further complicated by conflicts of commercial interests may involve us in war. At any rate, it is unwise to leave matters to chance and derogatory to our self respect to look for peace as a matter of forbearance.

The argument as to the adequacy of a citizen soldiery rising to the occasion with a burst of patriotism is not supported by history. Unpreparedness brought the disgrace of a captured national capital and of a campaign of little credit in the War of 1812. It protracted the Civil War from a conservative estimate of 90 days to four years and multiplied the cost in lives and money many times more. The Mexican War was on a numerically small scale, fairly commensurate with our standing army, was mainly fought by well trained troops and showed the results of careful training. The Spanish War, even though on a small scale showed up our unpreparedness, both military and more particularly from the standpoint of military hygiene. The Revolution is, therefore, the only war on which the theory of spontaneous rallying to the support of the country has been seriously based. It is only the most superficial consideration of history that warrants such a moral. Practically every man and boy in the colonies was proficient in the use of such weapons as were in vogue at the time. A reserve of veterans from the numerous colonial fights had been available during practically the whole colonial period. Organization and drilling had been carried on for several years. The revolution had been recognized as imminent, though perhaps not regarded as so serious and radical as it proved to be, for over a century, and indeed, one colony had started it almost exactly a century previously. With due regard for differing conditions of communication, transportation of troops, provisions of heavy armament, extant means of feeding and caring for the health of the men, and, most important of all the conceivable number of invading forces, our Revolutionary forces were in a state of preparedness far beyond anything demanded at present except by extreme militarists.

But we wish to urge military preparedness, more from the standpoint of peace than of war. The wonderful efficiency of Germany may be explained by the fact that she has given Kultur an active instead of a passive meaning. By a thoroughly systematized plan of economy and efficiency, she developed the most powerful army known to history and, up to the declaration of war, without detracting from the prosperity and strength of the nation. Dr. Lucien Howe has recently written of the advantages of military training, particularly physical and as developing strength of character. (See Abstracts). This is a phase of militarism that would pay for itself, if none of the men ever carried a gun. It is one, too, in which the medical profession should be especially interested. We have already pointed out that it is not impossible that the development of wireless electricity may eliminate the use of present explosives from warfare. At any rate, wars of the immediate future will be fought largely with gasoline, good roads, anti-typhoid vaccination, sanitary latrines, machines for transportation and excavation, and many other material things and methods which have no direct bearing on the destruction of life but which rather conduce to the welfare of society whether war comes or not. Preparedness for war involves a long period of careful planning. We may well make a beginning with those items which do not necessarily tempt to war and which will count for human health and efficiency and general prosperity and convenience.

